# Silicon isotopes in Arctic and sub-Arctic glacial meltwaters: the role of subglacial weathering in the silicon cycle

**DOI:** 10.1098/rspa.2019.0098

**Published:** 2019-08-14

**Authors:** Jade E. Hatton, Katharine R. Hendry, Jonathan R. Hawkings, Jemma L. Wadham, Sophie Opfergelt, Tyler J. Kohler, Jacob C. Yde, Marek Stibal, Jakub D. Žárský

**Affiliations:** 1 School of Earth Sciences, University of Bristol, Bristol, UK; 2 School of Geographical Sciences, University of Bristol, Bristol, UK; 3 National High Magnetic Field Lab and Earth, Ocean and Atmospheric Sciences, Florida State University, Tallahassee, FL, USA; 4 German Research Centre for Geosciences GFZ, Potsdam, Germany; 5 Earth and Life Institute, Environmental Sciences, Université Catholique de Louvain, L7.05.10, 1348, Louvain-la-Neuve, Belgium; 6 Department of Ecology, Faculty of Science, Charles University, Prague, Czechia; 7 Stream Biofilm and Ecosystem Research Laboratory, School of Architecture, Civil and Environmental Engineering, École Polytechnique Fédérale de Lausanne, 1015 Lausanne, Switzerland; 8 Department of Environmental Sciences, Western Norway University of Applied Sciences, Sogndal, Norway

**Keywords:** subglacial weathering, silicon isotopes, silicon cycle, glaciers and ice sheets

## Abstract

Glacial environments play an important role in high-latitude marine nutrient cycling, potentially contributing significant fluxes of silicon (Si) to the polar oceans, either as dissolved silicon (DSi) or as dissolvable amorphous silica (ASi). Silicon is a key nutrient in promoting marine primary productivity, contributing to atmospheric CO_2_ removal. We present the current understanding of Si cycling in glacial systems, focusing on the Si isotope (δ^30^Si) composition of glacial meltwaters. We combine existing glacial δ^30^Si data with new measurements from 20 sub-Arctic glaciers, showing that glacial meltwaters consistently export isotopically light DSi compared with non-glacial rivers (+0.16‰ versus +1.38‰). Glacial δ^30^Si_ASi_ composition ranges from −0.05‰ to −0.86‰ but exhibits low seasonal variability. Silicon fluxes and δ^30^Si composition from glacial systems are not commonly included in global Si budgets and isotopic mass balance calculations at present. We discuss outstanding questions, including the formation mechanism of ASi and the export of glacial nutrients from fjords. Finally, we provide a contextual framework for the recent advances in our understanding of subglacial Si cycling and highlight critical research avenues for assessing potential future changes in these environments.

## 1. Review of active processes within the Si cycle

Physical and chemical weathering in subglacial environments results in the export of key nutrients to the ocean that are required for marine primary production [1–13]. Understanding the silicon (Si) cycle is key to the consideration of the carbon cycle, because of the coupling between the two. Silicon is a vital nutrient for diatoms, siliceous algae, that account for approximately 40% of oceanic carbon export [14–16]. The Si cycle has been well described in recent years [17–21]. Broadly, there are two relatively distinct sub-cycles: continental and oceanic. Initially, dissolved Si (DSi) is liberated from silicate minerals through chemical weathering in the form of H_4_SiO_4_ [22,23]. The subsequent formation and burial of marine carbonates results in the sequestration of atmospheric CO_2_, hence silicate weathering is also an important sink of CO_2_ on geological time scales [21]. Equations (1.1)–(1.3) outline examples of the silicate weathering reactions that occur, including those hypothesized under glaciers.

Equation (1.1), weathering of anorthite:

1.1 $$\begin{array}{rl} \text{CaA}{\text{l}}_{2}\text{S}{\text{i}}_{2}{\text{O}}_{8(s)}+2\text{C}{\text{O}}_{2(aq)}+8{\text{H}}_{2}{\text{O}}_{\left(l\right)} & \to {\text{Ca}}_{\left(aq\right)}^{2+}+2\text{Al}{\left(\text{OH}\right)}_{3(s)}+2{\text{H}}_{4}\text{Si}{\text{O}}_{4(aq)}+2{\text{HCO}}_{3(aq)}^{-},\\ {\text{Ca}}_{\left(aq\right)}^{2+}+2{\text{HCO}}_{3(aq)}^{-} & \to \text{CaC}{\text{O}}_{3(s)}+{\text{H}}_{2}\text{C}{\text{O}}_{3(aq)}. \end{array}\}$$

Equation (1.2), silicate hydrolysis of albite:

1.2 $$\text{2NaAlS}{\text{i}}_{3}{\text{O}}_{3(s)}+9{\text{H}}_{2}{\text{O}}_{\left(l\right)}+{\text{2H}}_{\left(aq\right)}^{+}\to \text{A}{\text{l}}_{2}\text{S}{\text{i}}_{2}{\text{O}}_{5}{\text{(OH)}}_{\text{4}(s)}+\text{4}{\text{H}}_{\text{4}}\text{Si}{\text{O}}_{\text{4(}aq\text{) }}.$$

Equation (1.3), congruent weathering of pyroxene:

1.3 $$\text{Ca}(\text{Mg},\text{Fe})\text{S}{\text{i}}_{2}{\text{O}}_{6(s)}+4{\text{H}}_{\left(aq\right)}^{+}+2{\text{H}}_{2}{\text{O}}_{\left(l\right)}\to {\text{Ca}}_{\left(aq\right)}^{2+}+\frac{{\text{Mg}}_{\left(aq\right)}^{2+}}{{\text{Fe}}_{\left(aq\right)}^{2+}}+2{\text{H}}_{4}\text{Si}{\text{O}}_{4(aq)}.$$

The resultant DSi is transported to the oceans predominantly via rivers and groundwater [17], and as biogenic phases formed by the secretion of silica (i.e. silicon dioxide) by primary producers, such as diatoms and higher plants. We now have improved budgets of numerous aspects of the Si cycle, which can be used when attempting to estimate input and output fluxes to the ocean [17,18]. Riverine fluxes comprise the main input of Si into the oceans (7.3–8.1 Tmol yr^−1^, [17,19]), but other inputs also include aeolian dust (0.3 Tmol yr^−1^), groundwater (0.6 Tmol yr^−1^) and hydrothermal fluids (0.6 Tmol yr^−1^) according to Cornelis *et al.* [24] and Frings *et al.* [17]. However, data from glacial environments are still rare, which could have important implications especially when quantifying the riverine inputs to high-latitude oceans.

Glacier melting has accelerated over the past two decades, with Greenland Ice Sheet (GrIS) freshwater fluxes into the Irminger Basin increasing by 50% in less than 20 years [25]. Similarly, overall GrIS discharge has accounted for approximately 1000 km^3^ of freshwater discharge into the ocean per year between 2000 and 2010 [26–29], with rapid, nonlinear increases in run-off expected in the future [29]. Increased melting and resultant changes in hydrological pathways and subglacial water sources are likely to have an impact on dissolved nutrient export [1] and long-term climate modulation through the global carbon cycle [30]. Glacial weathering also results in the production of finely ground glacial flour, meaning a large fraction of nutrients exported in glacial meltwaters are associated with suspended sediments [1,31]. However, the future of suspended sediment fluxes from glacial environments is uncertain [32], and may depend on changes to surface melting, induced ice motion and subglacial hydrological drainage [33,34].

Knowledge of current glacial biogeochemical processes and their drivers within the subglacial environment is crucial to our understanding of how glacial nutrient fluxes and the resultant impact on downstream ecosystems may respond under climatic warming scenarios.

The purposes of this paper are as follows.

(i) Present new DSi and amorphous silica (ASi) data (both concentrations and Si isotopic composition) from a range of Arctic and sub-Arctic glaciers.
(ii) Compare the isotopic composition of Si from glacial meltwaters with that from non-glacial rivers and consider the main hypotheses for the mechanisms driving the differences between the measured values, in the context of subglacial weathering processes.
(iii) Discuss the potential implications of these findings for interpretations of the past global Si cycle, and future scenarios as glacial environments respond to anthropogenic climatic change.

This work builds on reviews of continental Si cycling by Opfergelt & Delmelle [20] and Frings *et al.* [17] by considering the role of glacial systems on terrestrial fluxes of Si into the oceans and glacial weathering influence on Si isotope composition. It will focus on Arctic and sub-Arctic glacial systems, as this is where studies into δ^30^Si composition of glacial rivers have been conducted so far, and our understanding of the weathering processes and fluxes from these environments is most complete. Unfortunately, there are no δ^30^Si data from Antarctica and temperate alpine glaciers, preventing this review from conducting a global assessment of δ^30^Si composition from glacial rivers. However, the combination of new data from 20 Arctic and sub-Arctic glaciers and the existing data with published literature enables a useful consideration of glacial δ^30^Si composition, and the potential subglacial drivers and impact upon the wider Si cycle.

### (a) Silicon isotope fractionation in low-temperature Earth surface processes

Silicon isotopes are a powerful tool for understanding geochemical processes within modern and past environments [17]. Silicon has three stable isotopes, ^28^Si, ^29^Si and ^30^Si, with respective abundances of approximately 92.18%, 4.68% and 3.15% [35]. The Si isotopic composition is reported in delta notation in terms of ^29^Si or ^30^Si. Here, we will report data in terms of δ^30^Si, according to equation (1.4),

1.4 $$\delta^{30}\text{Si}=\left[\frac{({}_{}^{30}\text{Si/}{}_{}^{28}\text{Si})\text{sample}}{({}^{30}\text{Si/}{}^{28}\text{Si})\text{NBS}28}-1\right]\times 1000.$$

Samples are reported against a known reference standard, most commonly NBS28 (RM 8546), which is a quartz standard distributed by the National Institute of Standards and Technology (NIST). Fractionation occurring during Earth surface processes, as a result of both kinetic and equilibrium effects, is mass dependent such that values of δ^30^Si are related in a predictable manner to δ^29^Si, δ^29^Si ∼ 0.5 × δ^30^Si [17,36]. The fractionation factor is reported as *α* (equation (1.5)) and is calculated as the fractionation from the substrate (*A*) to the product (*B*). Since the numerical value of *α_A_*_−*B*_ is usually very close to 1, it is also expressed in terms of per mil notation (ε, equation (1.6)) [17,18],

1.5 $$\alpha_{A-B}=\frac{{{(}^{30}\text{Si}:{}^{28}\text{Si})}_{A}}{{{(}^{30}\text{Si}:{}^{28}\text{Si})}_{B}}$$

and

1.6 $$\varepsilon =1000\cdot (\alpha_{A-B}-1).$$

Natural processes occurring at the Earth surface often comprise a complex chain of reactions, and quantification of the contribution of each step to the overall stable Si isotope fractionation is challenging. However, observations reveal that there is some common isotopic fractionation behaviour during low-temperature biogeochemical processes. When silicate minerals are weathered, forming secondary weathering products, the newly formed solids will be enriched with the lighter isotopes, compared with the parent rock and the residual waters which will be isotopically heavier [17,37]. Similarly, biological processes also result in the fractionation of Si. The uptake of silicic acid by primary producers, such as plants and diatoms, and/or the conversion of this to biogenic silica (BSi) preferentially incorporates the lighter isotopes into the newly formed BSi, leading to residual waters becoming isotopically heavy [38–40].

The magnitude of fractionation is dependent on numerous environmental factors, with laboratory studies attempting to quantify fractionation factors producing a large range of values. Experiments of ASi precipitation below 50°C by Geilert *et al.* [41] found a range of fractionation factors from +0.1‰ to −2.1‰, with lower temperatures producing the largest fractionation factors. However, they also found that these fractionation factors were influenced by mineral surface area, saturation state and flow regime, making it challenging to apply their findings to the natural environment. In comparison, precipitation experiments by Oelze *et al.* [42] found no fractionation during the formation of almost pure Si solids, but experiments with high Al/Si ratios produced fractionation factors of up to −5‰, with regimes beginning as unidirectional but shifting towards steady state once re-dissolution began. These two laboratory experiments present advances in our understanding of the magnitude of fractionation in low-temperature environments, but also highlight the difficulties in applying laboratory findings to real-world data owing to the complexity of the systems.

Rivers integrate the complex biogeochemical reactions occurring during weathering and biological utilization within their catchments, capturing the overall isotopic fractionation during these low-temperature processes [38,43–46]. The mean isotopic composition of dissolved riverine Si (δ^30^Si_DSi_) is currently reported as +1.25‰, from at least 557 individual measurements [17]. The isotopic composition of riverine waters can be linked to chemical weathering processes [47–49], as measurements show that the secondary weathering products are isotopically lighter than the DSi of river waters. Sutton *et al.* [18] conceptualized the processes as a mass balance between ‘kinetically limited’ and ‘supply limited’ to show how Si fluxes and isotopic composition are influenced by a balance of weathering and physical erosional processes. Kinetically limited systems are those where there is an excess of fresh material to be weathered but weathering fluxes are limited by external conditions, such as temperature. This best describes a subglacial environment, whereas supply-limited systems are those constrained by the supply of fresh weatherable material and thus almost all of the material is exported from the catchment as a dissolved flux [18].

**Figure 1.**
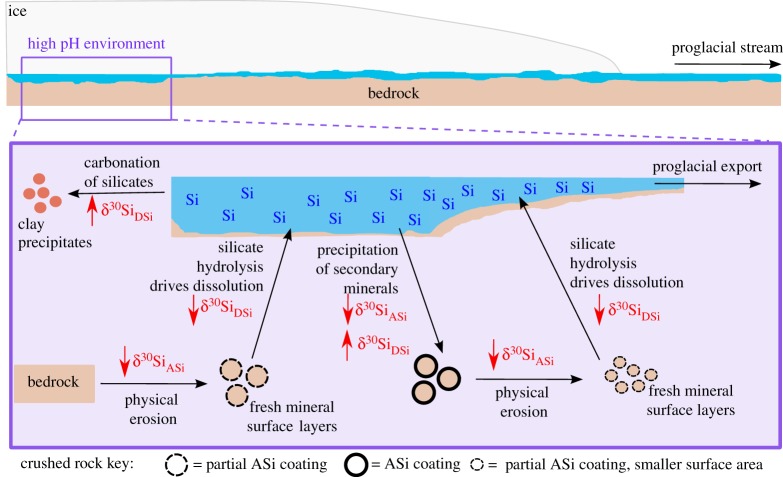
Conceptual model tracing subglacial processes impacting upon dissolved silicon isotope composition (δ^30^Si_DSi_) and silicon isotope composition in amorphous silica (δ^30^Si_ASi_). The hypothesized change in δ^30^Si composition as a result of each subglacial processes is denoted by red arrows. The pH of the environment is high as a result of alkalinity from silicate hydrolysis reactions. (Online version in colour.)

To date, two key observations have been made when considering the δ^30^Si_DSi_ of rivers [17,18]. First, the δ^30^Si_DSi_ composition of rivers is almost always higher than the parent material from which it has derived. Second, there is often a pronounced seasonal variation of approximately 0.5–1.0‰, with the lighter compositions linked to times of higher riverine discharge [17]. One important exception exists: the δ^30^Si_DSi_ composition of glacial meltwaters within proglacial rivers does not fit the observation that the δ^30^Si_DSi_ composition will be higher than the parent material [50]. The benefit of using Si isotopes in glacial systems to trace subglacial weathering processes is the lack of interference from primary productivity within proglacial streams. Proglacial rivers are extremely turbid owing to the high suspended sediment loads, resulting in an unfavourable environment for primary producers such as diatoms. Therefore, the Si isotope composition measured in these environments can be attributed to weathering processes, without the requirement of coupling the measurements with other isotope systems to deconvolve biological effects; e.g. Pogge von Strandmann *et al.* [38]. Understanding the drivers behind isotopic fractionation in subglacial environments may therefore provide insight into subglacial weathering regimes which are inherently different from those driving the δ^30^Si_DSi_ composition of non-glacial rivers (as detailed further in §1b, figure 1).

### (b) Understanding subglacial weathering processes

In order to predict the fluxes of key nutrients exported from glacial environments in the future we need to understand the mechanisms driving them, especially considering the complex nature of subglacial weathering processes, which are dependent upon subglacial hydrological evolution, bedrock lithology, and microbial community abundance and composition. As a result, hydrochemistry in proglacial rivers is often very different from that in non-glacial rivers, so the proportions of typical riverine weathering reactions cannot necessarily be applied in these environments. For example, initial studies of glacierized catchments, which focused on small glaciers, found glacial meltwaters to be lower in Si than non-glacial rivers [51–53]. More recent compilations of data from various glacierized catchments, including much larger catchments, also showed that dissolved K^+^ :  Na^+^ ratios (indicative of silicate mineral dissolution) are higher from glacial environments than data from the world's largest rivers, and ratios of Ca^2+^ :  Na^+^ and $\text{S}{{\text{O}}_{4}}^{2-}:\;\text{N}{\text{a}}^{+}$ are also significantly different [54], despite lower Si in glacial rivers. Chemical weathering yields from glacierized catchments (indicated by cation loading) have been shown to be above average when considering global rivers, because of the high run-off and physical erosion from these environments [54]. Conceptual models of the subglacial weathering processes that result in these distinct proglacial river chemistries have also been described previously [55–58].

Subglacial environments have complex hydrological systems, which develop seasonally [57,59]. This heterogeneity results in some areas of the bed with isolated waters and inefficient drainage pathways (i.e. tortuous flow paths), and other areas with efficient drainage pathways and short residence time waters [57]. This leads to differences in saturation state of primary and secondary minerals, and varying dissolution/precipitation reactions. Evolution and connection of hydrological flow paths and the introduction of supraglacial water will also impact water residence times and biogeochemical properties [60]. The efficiency of the subglacial pathways changes through an ablation season, which is true of small and large glacial systems [57,61,62], although predicting when changes will occur is difficult because of the complex nature of the subglacial system and inter-annual variability [63]. Such complex and temporally heterogeneous processes are likely to result in variations in isotopic fractionation in these environments.

Some of the first in-depth geochemical studies of subglacial weathering reactions took place at Haut Glacier d’ Arolla in the Swiss Alps [57,64]. Here, the subglacial waters, sampled via boreholes, were split into three categories (Types A, B and C), demonstrating the heterogeneity within the subglacial environments. Type A waters were the most concentrated in major ions, predicted to form in hydrologically inefficient areas. Type B waters were the most common waters, derived from more efficient parts of the drainage system, with chemical composition likely to be influenced by water residence time. Type C waters were the most dilute but had high turbidity, potentially representative of channel marginal zones [57]. Each water type was dominated by distinct geochemical reactions, for example waters in inefficient distributed systems were dominated by coupled sulfide oxidation--carbonate dissolution (equation (1.7)), whereas samples from more efficient pathways had higher DSi, potentially as a result of high pH (due to carbonate or silicate hydrolysis (equations (1.8) and (1.2)) promoting Si dissolution,

1.7 $$\begin{array}{rl} & 4\text{Fe}{\text{S}}_{2(s)}+16\text{C}{\text{a}}_{1-x}(\text{M}{\text{g}}_{x})+15{\text{O}}_{2(aq)}+14{\text{H}}_{2}{\text{O}}_{\left(l\right)}↔\\ & \;16(1-x){\text{Ca}}_{\left(aq\right)}^{2+}+16x{\text{Mg}}_{\left(aq\right)}^{2+}+16{\text{HCO}}_{3(aq)}^{-}+8{\text{SO}}_{4(aq)}^{2-}+4\text{Fe}{\left(\text{OH}\right)}_{3(s)} \end{array}$$

and

1.8 $$\text{C}{\text{a}}_{1-x}(\text{M}{\text{g}}_{x})\text{C}{\text{O}}_{3(s)}+{\text{H}}_{2}{\text{O}}_{\left(l\right)}↔(1-x){\text{Ca}}_{\left(aq\right)}^{2+}+x{\text{Mg}}_{\left(aq\right)}^{2+}+{\text{HCO}}_{3(aq)}^{-}+{\text{OH}}_{\left(aq\right)}^{-}.$$

It was previously assumed that chemical weathering beneath ice sheets would be insignificant, as the bed would be frozen and isolated from the atmosphere [65], also leading to the assumption that these environments would be devoid of life. However, subsequent studies of GrIS outlet glaciers show complex geochemical signatures, evolving over the melt seasons and with solute concentrations comparable to smaller alpine glaciers [3,4,50,56,66,67], with microbial signatures providing evidence for complex biogeochemical reactions [68–72]. Bacterial activity has been shown to impact the rate of dissolution of amorphous silica phases [73] and microbial processes play an important role in solute acquisition where the combination of unfrozen bed, suitable redox conditions and sufficient concentrations of carbon substrates and other nutrients permits the existence of an active subglacial ecosystem. Weathering regimes in large ice sheet systems appear to differ from smaller alpine glaciers, which could be a result of differences in water residence time, catchment size and drainage system dynamics. Wadham *et al.* [56] outline a predictive framework of subglacial chemical weathering processes by using data from a range of glacial systems of varying size, to demonstrate the role of ice mass and water residence time on the predominant geochemical reactions, and the chemical composition of exported meltwaters. Greater isolation and longer subglacial water residence times can occur in larger systems, which can lead to the enhancement of silicate dissolution due to calcite saturation or exhaustion of calcite minerals by prolonged weathering [56]. This is likely to be different from smaller glacial systems, where carbonate dissolution dominates, and saturation or exhaustion of calcite minerals is not observed to the same degree. This is true in spite of bedrock, as seen in many small glacial catchments (e.g. Engabreen, Haut Glacier d'Arolla and Lemon Creek Glacier [56,57,64,74]). In these systems silicate-rich bedrock exists but coupled carbonate dissolution--sulfide oxidation still dominates chemical weathering [56,74], owing to the exposure of trace carbonates from bedrock via physical erosion.

More recent work by Graly *et al.* [55] suggests that the response of subglacial chemistry to changing water residence times is varied, probably because of the complex controls exhibited from a wide range of glaciological features, and atmospheric gas (CO_2_ and O_2_) and sediment supply, which play an important role in spatial and seasonal characteristics of subglacial geochemistry. These authors use data from a range of glaciers to provide evidence that the abundance of atmospheric gas at the bed, primarily from surface melt [75], results in carbonation being the dominant subglacial process [55]. Freshly comminuted sediment is also important for subglacial weathering processes, as the availability of fresh mineral surfaces and trace reactive components impacts the proportion of easily dissolvable minerals (such as carbonates) within subglacial environments [55].

The hydrochemistry of the Watson River and specifically Leverett Glacier (LG), west Greenland, has been well studied owing to the large hydrologically active catchment size of LG (approx. 600 km^2^) and its potential as a modern-day analogue for past ice sheets [50]. Hindshaw *et al.* [67] found low Ca^2+^/Na^+^ molar ratios (approx. 0.6) at LG, with elevated Na^+^ and K^+^ concentrations over Ca^2+^ and Mg^2+^ indicative of enhanced silicate weathering [56]. There are also characteristics of subglacial drainage that are more prevalent in large ice sheet catchments than in small valley glaciers. A good example is surface melt-driven subglacial outburst events in Greenland. ‘Outburst events’ occur during the ablation season at LG, and result in the flushing of the subglacial system via the rapid drainage of supraglacial lakes to the bed [62]. These outburst events result in elevated discharge, suspended sediment, pH and electrical conductivity, indicating they are flushing previously isolated areas of the subglacial system where water residence times may be significantly longer than those in areas of the bed accessed regularly or well connected to the existing hydrological drainage system [1,62]. The elevated Na^+^ concentrations at LG coincide with outburst events during the melt season, which is consistent with the hypothesis of longer residence time waters with characteristics of enhanced silicate weathering. Hatton *et al.* [58] also found a switch from Ca^2+^ to Na^+^ as the dominant cation in the meltwater river at LG as the melt season progressed and silicate weathering became the predominant weathering pathway.

Understanding the complex subglacial processes beneath large glacial systems is challenging, because of the difficulties around access and sampling logistics. We have used the subglacial reaction frameworks published previously [55–58], combined with measurements of δ^30^Si composition in proglacial rivers, to produce a conceptual model of the subglacial processes impacting upon δ^30^Si composition in the subglacial system (figure 1), in order to highlight the most important processes driving the exported δ^30^Si composition. Figure 1 outlines the most likely subglacial weathering processes influencing δ^30^Si_DSi_ and δ^30^Si_ASi_ composition of glacial rivers, based upon current Si isotope measurements combined with hydrogeochemical data and concentrations of DSi, ASi and major ions. High physical erosion rates may be an important factor in determining δ^30^Si_DSi_ composition. Rock comminution results in fresh mineral surface layers enriched in isotopically light Si [76], potentially as ASi, however the formation mechanism for such is not well constrained, as discussed in §3c(ii). The dissolution of these mineral surface layers results in waters with an isotopically light δ^30^Si_DSi_ composition. If these areas of the subglacial environment are hydrologically connected with the main subglacial drainage system, then this signal is exported to the proglacial environment. We would therefore expect both small and large glacial catchments to export Si of isotopically light δ^30^Si_DSi_ composition, regardless of the dominant weathering regime, if we consider the relatively short-lived interactions between freshly comminuted surfaces and undersaturated meltwater, when hydrological drainage is efficient.

If we consider a large glacierized catchment then we can also consider the longer time-scale processes that could impact the δ^30^Si_DSi_ and δ^30^Si_ASi_ composition. We expect physical erosion and the dissolution of these isotopically light mineral surfaces to still be important in large systems when the subglacial hydrology is well developed, but we may also expect areas of subglacially stored waters with long residence times. In these long residence time waters it has been predicted that precipitation of secondary weathering products, such as ASi and potentially clays (discussed further in §3a), may occur as a result of supersaturation with respect to DSi (at least locally). Blackburn *et al.* [77] suggest that areas of low basal pressure result in the partial consumption of subglacial waters through local freezing, and cause solutes such as Si, Al, Fe and U to precipitate [77]. These secondary weathering products, such as ASi coatings, would be isotopically light, and subglacially stored waters would be enriched in heavier isotopes. However, if these areas of the bed then become hydrologically connected, further physical erosion and the addition of undersaturated waters during transport may occur. Therefore, these secondary weathering products may undergo redissolution, enriching the exported subglacial waters in isotopically light Si. This means that the δ^30^Si_DSi_ composition measured in proglacial rivers is always likely to be isotopically light, despite the potential for secondary mineral formation in isolated areas of the bed. This could potentially help to explain the isotopically light δ^30^Si_DSi_ composition during outburst events at LG [58], when we expect the export of long residence time waters.

In smaller catchments we would expect a dominance of carbonate weathering processes and therefore the formation of secondary weathering products such as ASi and clays would be potentially reduced. It is therefore likely that this precipitation–redissolution process would be less prevalent in smaller systems with shorter residence times, meaning we might expect the light δ^30^Si_DSi_ composition to mainly be driven by the interaction between freshly ground mineral surfaces and hydrologically connected undersaturated waters.

### (c) Silicon fluxes from glacial systems

Complex subglacial geochemical processes, especially beneath large glaciers draining ice sheets, have the potential to export previously underappreciated fluxes of DSi and dissolvable ASi [2,7]. At present, we are limited by the small number of field studies, limiting the inferences we can make on subglacial weathering processes and potential nutrient fluxes presently and in the future. Furthermore, these environments are spatially and temporally heterogeneous, as illustrated by the GrIS, from which 80% of the total sediment is exported by only 15% of Greenlandic rivers [78].

Increasing focus has been placed on quantifying the fluxes of key nutrients from glacial systems [1,2,5,6,13,79,80] and understanding the weathering processes driving these fluxes [7,55–57,67,81–84]. Meltwaters from the GrIS contain DSi and ASi that may constitute significant fluxes of bioavailable Si into fjords and polar oceans. Meire *et al.* [5] estimated the export of DSi as 0.02 Tmol yr^−1^, when upscaling data from two fjord systems and a range of glacial meltwater rivers. While this estimate is large relative to other nutrients, particulate Si associated with suspended sediment may increase this flux further. Hawkings *et al.* [2] considered both the dissolved and amorphous phases, resulting in a larger estimate of mean Si flux by an order of magnitude for the GrIS of 0.20 (0.06–0.79) Tmol yr^−1^, approximately 50% of the input from Arctic rivers, with ASi accounting for greater than 95% of this flux. Dissolution experiments have shown this ASi to be highly soluble in seawater [2], consistent with laboratory studies of both biogenic and synthetic amorphous phases [85]. Therefore, it is likely that this Si is either directly or indirectly bioavailable (as DSi or ASi respectively) and could therefore stimulate the growth of diatoms and influence primary production. These findings are significant when considering the potentially high export of suspended sediments from the GrIS [86]; Overeem *et al.* [78] calculate that up to 1.28 Gt yr^−1^ of suspended particulate matter (SPM) from the GrIS is entering the surrounding oceans. This agrees well with estimates of SPM export from the GrIS derived from LG; Hawkings *et al.* [2] estimated a maximum GrIS SPM export of 1.7 Gt yr^−1^, with a more conservative mean estimate of 0.49 Gt yr^−1^. However, the extent to which dissolved nutrients and those associated with this SPM reach surface and open ocean waters may be limited by nutrient drawdown and burial within complex fjord systems (see §4).

The export of Si from glacierized catchments varies (electronic supplementary material, table S1) and this is likely to be a result of varying subglacial conditions and reactions, as described in §1b. The purpose of the compilation in this review paper is to demonstrate Si yields from various glacial systems measured thus far, and to highlight the potential glacial systems have for contributing to regional biogeochemical cycles through the export of Si. Silicon yields were calculated according to equation (1.9) and Si concentrations, discharge and catchment size were compiled from a range of published studies.

1.9 $$\begin{array}{l} \text{Si yield }(\text{ton}\;\text{k}{\text{m}}^{-\text{2}}{\text{a}}^{-1})=\frac{Q\times \;Q_{\text{wt}}\text{Si}}{\mathrm{Catchment}\;\mathrm{area}}\\ \text{Where:}\\ Q=\text{annual }\text{discharge }(l)\\ Q_{\text{wt}}\text{Si}=\text{discharge-weighted Si }\text{concentration}\\ \text{Catchment area}=\text{glacial catchment size }(\text{k}{\text{m}}^{2}) \end{array}$$

## 2. Methods to characterize Si in glacial meltwaters

**Figure 2.**
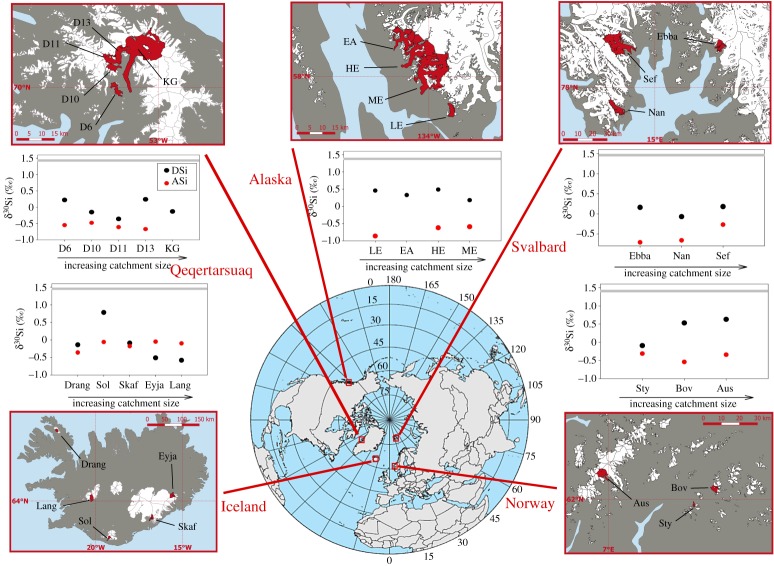
Location map of glacial rivers analysed for the silicon isotope (δ^30^Si) composition for dissolved (DSi) and amorphous (ASi) phases. The proglacial rivers of the glaciers highlighted in red in the five smaller maps were sampled for δ^30^Si composition. Clockwise from lower left; Iceland, Qeqertarsuaq (Disko Island), southeast Alaska, Svalbard and Norway. Graphs show the δ^30^Si_DSi_ (black) and δ^30^Si_ASi_ (red) composition measured for each glacier, organized by location. Error bars, mainly included in the symbol size, represent average external errors (0.08‰, 2 s.d.). Glaciers are organized by catchment size on each graph (acronyms defined in electronic supplementary material, table S2). The horizontal grey line represents the average non-glacial δ^30^Si_DSi_ composition compiled from published data (see figure 3 for references). (Online version in colour.)

To expand the existing dataset and provide a broader perspective into the δ^30^Si composition of glacial meltwaters, we present new observations from 20 glaciers from across the Arctic and sub-Arctic (electronic supplementary material, table S2; figure 2). New samples were collected from August 2015 to September 2017 from glaciers corresponding to one of five major regions (Qeqertarsuaq (Disko Island), Iceland, Norway, Alaska and Svalbard), and each glacier was spot sampled once due to logistical restrictions.

There are currently some differences in how the community collects and analyses samples for δ^30^Si composition, so we attempt to compile the range of methods currently in use (table 1), alongside describing the methods we employ. We highlight any differences and assess the potential implications of these discrepancies.

**Table 1.** Compilation of published methods for sample collection and storage for the analysis of silicon isotope composition from rivers.

		sample collection			
study	location	filter size (µm)	filter type	time before filtering	storage
Hawkings *et al.* 2018 [50]	Leverett, Greenland	0.45	cellulose nitrate	immediately	refrigerated in field
Opfergelt *et al.* 2013 [44]	Iceland	0.2	cellulose acetate	<24 h	
Georg *et al.* 2006 [43,100]	Switzerland	0.45	cellulose acetate	immediately	acidified with HCl
Georg *et al.* 2007 [49]	Iceland	0.2			acidified with HNO_3_
Ding *et al.* 2004 [101]	Yangtze River, China	not specified	not specified	<2 weeks	refrigerated
Ding *et al.* 2011 [102]	Yellow River, China	not specified	not specified	<24 h	
Cardinal *et al.* 2010 [46]	Congo River	0.2	cellulose acetate		refrigerated; acidified with conc. HNO_3_
Hughes *et al.* 2013 [103]	Amazon River	0.22	cellulose nitrate		

### (a) Filtration

There are various ways to prepare water samples for Si isotope composition analysis (table 1) and this could have potential implications when considering the data produced. There is currently no standard protocol for filtration (e.g. filter membrane size, commonly 0.45 µm or 0.22 µm, and/or filter type), although different membrane types are used to aid with sampling large volumes of water, or water high in suspended particulate phases. This lack of standardization has the potential for introducing bias if significant proportions of the total Si are held within colloidal or nanoparticulate size fractions, relative to the truly dissolved phase. Currently, both depth and screen membrane filters are used in the field. Depth filters have layers of randomly orientated fibres that make up the filter, and usually have a pore size rating determined from bubble point tests. Screen membrane filters (e.g. track etched polycarbonate) do not pass anything larger through than the stated pore size (measured by visual examination) by performing separation on the surface of the membrane. Morrison & Benoit [104] found that colloids of Fe, Al, Mn and organic carbon behaved differently when comparing the impact of filter clogging of different elements and that it cannot be assumed that a clogged filter is necessarily trapping the same proportion of colloidal phases of all elements. Depth filters can initially trap size fractions smaller than the specified filter size by adsorption but are less likely to clog and trap smaller particle sizes as filtration progresses. By contrast, screen filters clog more rapidly, with the effective pore size reducing progressively [105], and there is a strong correlation between increasing back pressure and decline of colloidal concentrations [104]. There is therefore a difficult balance between initially passing sample through the filter for a preliminary wash and to flush out any residual cleaning acid, filling any potential adsorption sites in the membranes and clogging up the filter membrane. This is especially critical with samples such as glacial meltwaters where the sediment load is high (commonly greater than 1 g l^−1^) and the effective pore size reduces quickly [105]. These issues present potential implications, considering that different filter membranes are used across research projects, which could alter the conclusions drawn about the truly dissolved δ^30^Si composition. The influence of filter membrane type on colloidal and nanoparticulate Si [2] has not yet been explored systematically in the field or experimentally.

### (b) Sampling methodology

The new data we present in this study (electronic supplementary material, table S2; figure 2) followed the methods reported in Hawkings *et al.* [50] and Hatton *et al.* [58]. Briefly, samples were collected using clean Nalgene bottles (high-density polyethylene—HDPE) and filtered immediately in the field through 47 mm 0.45 µm cellulose nitrate membrane filters (most similar in behaviour to the depth filters described above). The filtrate was kept refrigerated at 4°C in the dark until analysis. The filters were retained, kept refrigerated in the dark, and transported back to the laboratory and air dried in a laminar flow hood.

We ensured all samples reported in this study used the same filter types as those in previous studies of Greenlandic meltwaters [50,58], so we could confidently compare the data. However, other studies have used different filter types and different pore sizes (for example Opfergelt *et al.* [44] used 0.2 µm cellulose acetate filters), resulting in potential differences in what is defined as the truly dissolved phase when δ^30^Si_DSi_ composition is discussed.

### (c) Sample preparation for Si isotope analysis

Water samples for δ^30^Si_DSi_ were evaporated so that approximately 2 ml of sample had a concentration of approximately 2 ppm. This sample was subsequently passed through a pre-conditioned BioRad AG 50 W-X12 cation exchange resin for purification [100]. Suspended sediments from the filters (for δ^30^Si_ASi_) were extracted by the addition 0.2M NaOH for 40 min at 100°C. This extraction method was used as it targets the amorphous silica phase [106], which will complement ASi concentration data. Samples were then neutralized with 8N HNO_3_ and passed through a similar pre-conditioned BioRad cation exchange resin as for water samples.

Silicon isotope analysis was carried out in the Bristol Isotope Group laboratories (University of Bristol, UK) using a Thermo Scientific™ Neptune High Resolution Multicollector inductively coupled plasma mass spectrometer (MC-ICP-MS), using a standard-sample-standard bracketing procedure, using the international reference standard NBS-28 (NIST RM8546, purified quartz sand). Samples were also doped with intensity-matched Mg solution to correct for internal mass bias and 100 µl of 0.1M H_2_SO_4_ to account for potential anionic matrix mass bias [50,107].

## 3. Silicon isotope composition of glacial rivers

### (a) Theoretical isotopic composition of DSi

Glaciers are highly effective physical erosion agents. The process of rock comminution results in an abundant supply of fine-grained rock flour, exposure of trace reactive components in the bedrock, modification of mineral surfaces and subsequent chemical weathering, all of which are likely to influence the composition of meltwaters exported from the glacier. Our knowledge of the fractionation of Si isotopes during low-temperature surface processes allows predictions of the dissolved isotopic signature of glacial meltwaters. If indeed silicate mineral weathering is enhanced beneath large glacier systems, then we might expect a fractionation effect which enriches the residual meltwater in the heavier isotopes (due to preferential uptake of ^28^Si) during the formation of secondary weathering products, such as clays. The high pH within subglacial environments [7,58] and highly reactive nature of SPM favours secondary mineral precipitation, with reaction path models showing that subglacial water chemistries cannot be balanced when only considering dissolution processes [108]. However, subglacial clay formation is uncertain, with differing interpretations based upon proglacial meltwater and borehole chemistry [7,43,108,109]. For example, analysis of glacial rivers in west Greenland found that waters were undersaturated with respect to almost all clay minerals and X-ray diffraction showed no obvious signs of secondary clays [109], but it is unknown whether this is typical of all glacial catchments. δ^30^Si_DSi_ composition alone cannot currently be used to inform on clay formation, owing to repeated dissolution and reprecipitation cycles impacting upon isotopic fractionation and the differences in fractionation factors between different clay minerals [20,110,111]. The first smaller scale studies of δ^30^Si_DSi_ composition comparing glacial versus non-glacial riverine systems did not fully support the subglacial clay formation. Opfergelt *et al.* [44] analysed 18 river samples from glacierized catchments and non-glacierized catchments in Iceland and reported that the non-glacial rivers have a heavier δ^30^Si_DSi_ composition than the glacial rivers (+0.97 ± 0.31‰, compared with +0.17 ± 0.18‰). The δ^30^Si_DSi_ compositions measured in the non-glacial rivers were consistent with a supply-limited regime, with clay formation preferentially incorporating the light isotopes, resulting in heavier δ^30^Si_DSi_ composition. They inferred that the δ^30^Si_DSi_ compositions measured in the glacial rivers followed a so-called kinetically limited regime, with Si from the basaltic bedrock undergoing dissolution in relatively high pH conditions, but the extent of the weathering being insufficient to permit extensive clay mineral formation [44].

Consistent with this initial finding on rivers draining small Icelandic glaciers, Hawkings *et al.* [50] and Hatton *et al.* [58] also found a consistently light δ^30^Si_DSi_ composition when completing a seasonal study of subglacial run-off from LG. LG has a much larger hydrologically active catchment (approx. 600 km^2^) than most of the glaciers studied in Iceland, and the δ^30^Si_DSi_ composition was lighter on average than both the glacial Icelandic rivers and non-glacial rivers measured previously (discharge-weighted mean of −0.25‰). Variations in δ^30^Si_DSi_ composition alongside the major ion chemistry of run-off indicated a direct correlation between the δ^30^Si_DSi_ composition and the extent of silicate mineral weathering as inferred from ratios of Na^+^/Ca^2+^ and divalent/monovalent ions. By mid-melt season, when flow paths are longest, Hatton *et al.* [58] reported an isotopic signature of −0.52‰ at LG, which was the lightest δ^30^Si_DSi_ composition measured in running waters to date. Investigation of a smaller Greenlandic glacier found a δ^30^Si_DSi_ composition lighter than non-glacial river waters, but not as light as the δ^30^Si_DSi_ composition measured at LG, leading to the hypothesis that the larger catchment undergoes enhanced silicate mineral weathering, as a result of more isolated subglacial waters with longer residence times, driving the lighter δ^30^Si_DSi_ composition [58], as discussed further in §3b(ii).

### (b) New DSi isotopic data: what do we learn from an expanded dataset?

#### (i) Contribution of glacial rivers to global isotopic mass balance

In order to assess the isotopic composition of glacial meltwaters more widely, the present study incorporates new data from 20 Arctic and sub-Arctic glaciers (electronic supplementary material, table S3), which range in geographical location, bedrock lithology, catchment size, subglacial hydrological and weathering regime. Considering a wider range of proglacial rivers will help to quantify the average glacial melt-fed river δ^30^Si_DSi_ composition and assess the patterns and hypotheses drawn from the existing data.

**Figure 3.**
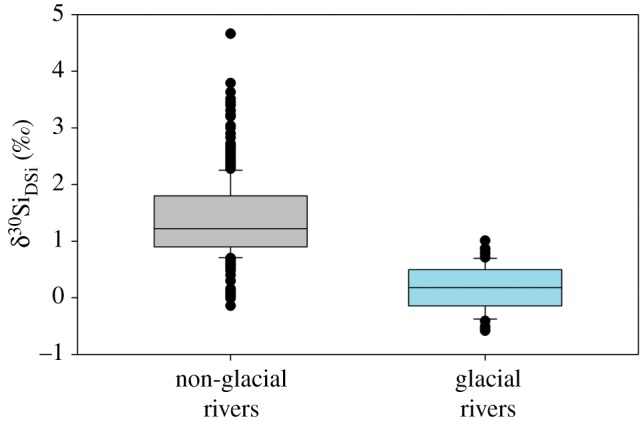
Box plot showing the global average dissolved silicon isotope (δ^30^Si_DSi_) composition from glacial and non-glacial rivers. Data compiled from existing published sources [44,49,50], from this study for glacial rivers (+0.16‰) and from published sources for non-glacial rivers (+1.38‰) [37,43–46,101–103,112–118]. Boxes represent upper and lower quartiles, with the median represented by the middle line. (Online version in colour.)

The δ^30^Si_DSi_ composition for all the glacial rivers measured is consistently lighter than the non-glacial average (figure 3). The sample from Langjökull (west Iceland) has the lightest δ^30^Si_DSi_ composition measured in running waters (−0.58‰), lower (but statistically within error) than the lightest value reported from LG in 2015 during a subglacial outburst event (−0.52‰ [50]). It is important to note that Langjökull was sampled during a heavy rainfall event, which could have had an impact on the meltwater chemistry. However, sampling was completed very close to the glacier terminus and light δ^30^Si_DSi_ composition was measured at other Icelandic glaciers (e.g. Eyjabakkajökull, −0.51‰) when weather conditions were dry and sunny.

The light δ^30^Si_DSi_ compositions measured from the new glaciers presented in this paper are consistent with findings from Georg *et al.* [49], Opfergelt *et al.* [44], Hawkings *et al.* [50] and Hatton *et al.* [58], indicating that subglacial weathering processes are the primary cause of the light δ^30^Si_DSi_ composition exported from these environments. The δ^30^Si_DSi_ composition from the Watson River in September 2017, which is fed by three outlet glaciers from the GrIS, including LG, was +0.31‰. This indicates that LG likely exports isotopically light DSi throughout the melt season, until shut-down of the hydrological system, which concurs with calculations by Hatton *et al.* [58]. This value is heavier than the δ^30^Si_DSi_ composition measured at LG during the peak melt season. This could potentially reflect mixing between waters of different origin, rather than a shift in subglacial weathering processes, as the Watson River is more influenced by non-glacial water sources than the LG proglacial river. Additionally, one of the outlet glaciers feeding the Watson River is Russell Glacier, which is a small outlet glacier with currently uncharacterized δ^30^Si_DSi_ composition. But we may expect the δ^30^Si_DSi_ composition from this glacier to be isotopically heavier than that of LG, based on the comparison of Greenland catchments by Hatton *et al.* [58]. Alternatively, assuming the hypothesis that isotopically light δ^30^Si_DSi_ composition is driven by mechanochemical reactions is correct, then we might expect to see an increase in δ^30^Si_DSi_ composition as glacial motion and rock grinding slows later in the season. This is consistent with the increasing δ^30^Si_DSi_ composition at the end of the monitoring period at LG in 2015 [50,58].

An updated database of non-glacial rivers provides a mean δ^30^Si_DSi_ composition of +1.38‰, compared with the previous estimates of +1.25‰ by Frings *et al.* [17] [37,43–46,101–103,110,112–118]. This compares with a mean δ^30^Si_DSi_ composition of glacial rivers of +0.16‰, based on the new glaciers reported in this study and glacial rivers published previously [44,49,50]. Note, in the absence of Si concentration data, discharge data and/or a seasonal record of δ^30^Si_DSi_ composition for many rivers we have reported a simple mean value.

#### (ii) Controls of δ^30^Si_DSi_ composition of glacial meltwaters

Statistical analyses of these data, such as regression model and principal component analysis, do not reveal any significant relationships to suggest an overriding process that is the main cause of the light Si isotopic composition in glacial meltwaters. This is likely to be due to the complex nature of subglacial processes and the temporal development of the hydrological system (and associated biogeochemical conditions) over a melt season [59,62,66,119]. Evidence from the limited number of Greenlandic catchments that have been monitored over a melt season suggests that changes in subglacial weathering regime, associated with hydrological evolution from an inefficient subglacial drainage system to an efficient channelized drainage system, influences the δ^30^Si_DSi_ composition of exported waters [58]. δ^30^Si_DSi_ composition generally decreased over the melt season, with the lowest δ^30^Si_DSi_ composition coinciding with outburst events at LG and the hydrological connection of the subglacial system at a smaller Greenlandic catchment, Kiattuut Sermiat (KS) [58]. We postulate that this pattern would also be seen in other glacial systems, had it been possible to monitor a larger range of glaciers over a longer time period rather than treating samples from all the glaciers as a single dataset.

There are currently various hypotheses for explaining the light δ^30^Si_DSi_ composition in glacial meltwaters. On the basis of evidence from Greenlandic glacial systems, Hawkings *et al.* [50] hypothesized that glaciers with a larger catchment size would export meltwaters with a lighter δ^30^Si_DSi_ composition. These authors suggest that the isotopically light amorphous mineral surface layers, which form through precipitation or leaching in a long residence system, subsequently undergo redissolution (due to high pH and undersaturation of waters with respect to ASi), resulting in a lighter δ^30^Si_DSi_ composition [50]. However, analysis of δ^30^Si_DSi_ composition from the wider range of glaciers presented in this study shows no significant relationship between δ^30^Si_DSi_ composition and catchment size, with glacierized catchment area ranging from 1.5 to 131 km^2^. This may be because of the challenges in assessing the size of the hydrologically active catchment area when the sample was taken from the proglacial river, and the lack of a clear relationship between catchment size and water residence time. It may also suggest that δ^30^Si_DSi_ composition of glacial meltwaters is not directly driven by catchment size, but potentially by a range of factors in addition to subglacial water residence times, such as physical erosion processes (e.g. mechanochemistry). We also postulate that differences in bedrock lithology would not result in significant variations in δ^30^Si_DSi_ composition [58], as high-temperature processes produce limited isotope fractionation and the δ^30^Si composition of crustal silicate rocks is relatively homogeneous [120,121].

**Figure 4.**
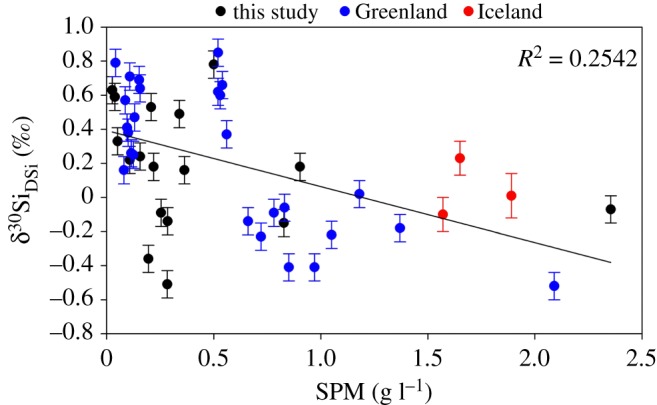
Relationship between suspended particulate material (SPM) concentrations and the dissolved silicon isotope (δ^30^Si_DSi_) composition of glacial meltwaters. Data are reported from glaciers measured in this study, Greenland [50,58] and Iceland [44]. Data have been trimmed to remove outliers (95th percentile), and the regression line has an *R*^2^ value of 0.2542. Error bars on data from this study represent the average external error of 0.08‰ (2 s.d.), based on triplicate measurements of a subset of samples. (Online version in colour.)

The very high rates of physical erosion in subglacial environments could also be considered the main driver for light δ^30^Si_DSi_ composition in glacial meltwaters, as discussed previously. Elevated physical erosion rates result in the formation of finely ground, reactive glacial flour and exposure of trace reactive minerals with very high surface areas [78,81,86,122,123]. This process is also a potential mechanism for formation of isotopically light ASi [76], as discussed in §3c(ii). There is likely to be preferential leaching of ^28^Si from these freshly ground mineral surfaces, resulting from kinetic fractionation, as demonstrated by dissolution experiments on Hawaiian basalts [76]. Dissolution conditions are favourable in the subglacial environment, despite the low temperatures, owing to the high pH (from hydrolysis of freshly ground carbonate and silicate minerals) and undersaturation of meltwaters with respect to ASi. There is some indication that higher suspended sediment concentrations are linked to lighter δ^30^Si_DSi_ composition in glacial meltwaters, with the lightest δ^30^Si_DSi_ values measured either during the outburst events at LG (categorized by SPM of 1–2 g l^−1^) or from Langjökull (where SPM was extremely high, at approx. 47 g l^−1^; figure 4). However, we have chosen to remove the SPM data from Langjökull from our analysis as this site was sampled during a very heavy rainfall event, and therefore the measured SPM is not likely to be representative of baseflow conditions.

The role of permafrost in modifying δ^30^Si composition of glacial streams has not been considered for the data presented in this review paper as all of the rivers have been sampled close to the glacier front. However, the impact of weathering reactions within permafrost active layers should not be overlooked if river samples are taken further from the subglacial outflow. The impact of permafrost on riverine δ^30^Si composition is relatively understudied at present but evidence suggests that seasonal changes in δ^30^Si composition of streams occur as a result of seasonal permafrost changes [110,115,124]. For example, the δ^30^Si_DSi_ composition of the Lena River decreases from winter to summer. This is likely to be due to the dominant winter Si source being from intrapermafrost layers with enhanced clay formation, whereas the summer Si is sourced from permafrost upper active layers where dissolution of silicate minerals and phytoliths occurs [110]. Therefore, it is important to consider the potential role of permafrost on Si flux from high-latitude rivers and upon the ‘glacial’ riverine δ^30^Si_DSi_ composition, especially under climatic warming scenarios and the predicted associated permafrost thawing.

### (c) Glacially derived amorphous silica

#### (i) Contribution of glacial amorphous silica to global budgets

The export of Si from glacial systems is dominated by the reactive ASi fraction, with the mean ASi concentrations in glacial rivers being significantly higher than from other riverine systems (e.g. mean ASi concentration of 392 µM from LG [2]). It is therefore important to investigate the isotopic composition of glacial ASi and improve our understanding of the formation and eventual fate of this fraction. Hawkings *et al.* [50] presented the first data on the δ^30^Si_ASi_ composition of riverine waters, analysing suspended sediments over a melt season from LG. This was followed by a study comparing two catchments, LG and KS in Greenland [58]. δ^30^Si_ASi_ composition in Greenland was light and relatively uniform for both catchments, consistently approximately 0.2‰ lighter than the bulk bedrock measurements of the catchments. δ^30^Si_ASi_ composition was generally lighter than the δ^30^Si_DSi_ composition (by 0.02 to +0.82‰ at LG and by 0.68 to +1.27‰ at KS), except for outburst events at LG, when the δ^30^Si_DSi_ composition was lighter by 0.37‰.

**Figure 5.**
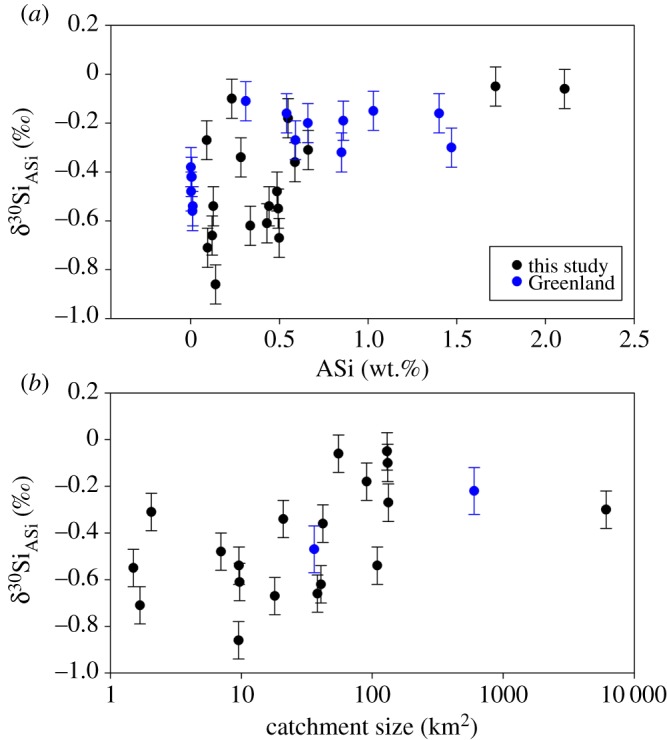
Relationships between catchment size and amorphous Si (ASi, wt.%) and the silicon isotope (δ^30^Si_ASi_) composition in suspended particulate matter of the new glacial meltwaters analysed in this study and from Greenland [58]. Error bars represent an average external error of 0.08‰ (2 s.d.), based on triplicate measurements of standards and a subset of samples, except for the Greenlandic samples in (*b*). These datapoints represent the discharge-weighted mean δ^30^Si_ASi_ composition from measurements over the ablation season and error bars represent the range of values measured over the season. (Online version in colour.)

The new glaciers presented in this study (with the exclusion of Langjökull, owing to the extreme SPM mentioned previously) have a δ^30^Si_ASi_ composition in the range of −0.05‰ to −0.86‰, with an SPM normalized average of −0.27‰. As no discharge data are available for the glaciers sampled, we used the suspended sediment concentration (in g l^−1^) to weight the δ^30^Si values, as this is the only common parameter available from all glaciers measured to enable a weighted mean. The SPM varies from 0.01 to 2.35 g l^−1^ with an average of 0.39 g l^−1^, and an average ASi concentration of 0.5 dry wt.% (0.09--2.1 dry wt.%). This is similar to the current mean ASi of global rivers (0.6 wt.% [17]). The average δ^30^Si_ASi_ composition of these new glacierized catchments is similar to the discharge-weighted (*Q*_wt_) mean δ^30^Si_ASi_ composition of a large Greenlandic catchment, LG (−0.22‰). Kiattuut Sermiat had a lighter *Q*_wt_ mean δ^30^Si_ASi_ composition of −0.47‰ [58], which is still within the range of values measured across these other glaciers. It is difficult to directly compare these values as the averages are not calculated with the same weighting (discharge-weighted mean versus weighted by suspended sediment concentration). There is no statistically significant relationship between δ^30^Si_ASi_ composition and SPM (*R*^2^ = 0.0048, *p* = 0.5278) when looking at the dataset as a whole, however there appears to be a general increase in δ^30^Si_ASi_ composition as catchment area and ASi concentration increase. Whilst these relationships are not statistically significant (*R*^2^ = 0.4149, *p* = 0.0657 and *R*^2^ = 0.3809, *p* = 0.0508, respectively; figure 5) they provide an interesting trend, and the data are complicated by lack of seasonal catchment records, which is likely to be causing the lack of a significant relationship.

The range of δ^30^Si_ASi_ compositions measured across these glaciers is also consistent with data presented from non-glacial rivers. Ding *et al.* [101] report an average δ^30^Si composition of SPM in the Yangtze River of −0.34‰ (with a range of 0‰ to −0.7‰) and Ding *et al.* [102] present an average δ^30^Si composition of −0.02‰ (0.3‰ to −0.4‰) for SPM in the Yellow River. However, it must be considered that these values are calculated from the total SPM, using total digests, which probably results in the inclusion of other clay minerals rather than a measurement of only the ASi phases. It is also likely that the δ^30^Si composition of SPM in these systems is significantly impacted by phytolith contributions, owing to the variable range of δ^30^Si composition that phytoliths exhibit [18,101,125]. None of the ASi within the highly turbid proglacial rivers measured is expected to be biogenic; the low temperatures, high SPM loads and subglacial origin indicate the environment is not conducive for primary producers.

Assuming simple mass balance, glacial δ^30^Si_ASi_ composition should be heavier than that in non-glacial rivers to offset the light δ^30^Si_DSi_ composition of these meltwaters. However, the Si pool in subglacial systems is so large that the export of isotopically light DSi does not have a significant impact upon the overall isotopic composition of the ASi reservoir. A simple fractionation model illustrates that less than 5% of the measured ASi is needed to undergo dissolution to produce the lightest δ^30^Si_DSi_ composition (−0.56‰ at LG [58]).

#### (ii) Controls of δ^30^Si_ASi_ composition from glacial sources

High-resolution transmission electron microscope photomicrographs show the presence of amorphous silica nanostructures in SPM from Greenland and iceberg-entrained debris [2]. The origin of ASi from glacial environments is not yet constrained, making it challenging to assess the Si isotope fractionation process during its formation. However, we can assume that the light δ^30^Si_ASi_ composition results from low-temperature fractionation associated with the enhanced physical and chemical weathering that occurs in subglacial systems. Two chemical weathering hypotheses for the formation of ASi have been proposed: the leached surface layer hypothesis and the dissolution–reprecipitation mechanism [2]. The leached surface layer hypothesis describes the dissolution of weakly bonded ions, resulting in the formation of amorphous crusts enriched in less soluble ions, such as silicon. The dissolution–reprecipitation hypothesis predicts the dissolution of the finely ground mineral surface layer and then the formation of ASi through the reprecipitation of silica due to supersaturation at the grain boundary [2,126].

A separate hypothesis is based on mechanochemical reactions from glacial bed grinding, whereby ASi is generated from a disturbed surface layer. These physical processes impact the structure of mineral surfaces [2,68], and result in the formation of surficial amorphous layers, with structural changes, lattice distortion and associated chemical changes [127,128]. Preferential dissolution of ^28^Si from these freshly ground mineral surfaces occurs due to kinetic fractionation [76], driving the system towards lighter δ^30^Si_DSi_ compositions by around −1‰.

Both physical and chemical weathering hypotheses for the formation of ASi result in isotopically light ASi, so analysis of SPM using Si isotopes alone will not help to distinguish the most likely formation mechanism. Photomicrographs of ASi nanostructures in sediments from Greenland and iceberg-entrained debris show that the ASi is mainly associated with the edges of other material, and electron diffraction spectroscopy indicates that less soluble elements such as Fe and Al are incorporated into its nanostructure [2]. While this means its formation could be a result of both aluminosilicate mineral weathering and/or mechanical grinding, novel high-resolution microscopic and spectroscopic techniques to investigate these structures and associated elemental characteristics may provide a way to distinguish between subglacial chemical and physical weathering processes in the future. Furthermore, it may be useful to conduct controlled laboratory experiments to mimic the subglacial system. For example, erosional processes could be simulated through rock crushing and dissolution could be traced using a combination of isotopic analyses and major ion concentrations and composition.

## 4. Wider impact of glacial Si

Glaciers have been associated with high nutrient fluxes, including Si in dissolved and dissolvable amorphous forms [2]. However, an important outstanding question is how much of this exported material reaches the open ocean, especially from the GrIS, where complex fjord networks exist.

### (a) Fjords as conduits of Si to the marine system

Upwelling of marine-sourced nutrients has generally been considered to dominate inputs to high-latitude fjords, both along the West Antarctic Peninsula [129] and in Greenland fjords [130]. However, glacially exported nutrients might have an important impact on biogeochemical cycles within fjord systems [2,13,131–133]. Furthermore, upwelling marine waters are likely to have been modified by inputs (e.g. of Si and Fe) from shelf sediments, which are composed of recently deposited glacially derived material [134–136].

In Greenland, Si concentrations are elevated in fjords in front of both land-terminating and marine-terminating glaciers as a result of meltwater input [5,137]. Young Fjord (with melt input from a land-terminating glacier) displayed a dominance of diatoms over other phytoplankton species, probably because of the increased availability of DSi from glacial meltwaters [5]. In Godthåbsfjord (with inputs from marine- and land-terminating glaciers), the upwelling of subglacial discharge led to sustained phytoplankton blooms with high Si : N ratios, resulting in a diatom-dominated summer phytoplankton assemblages (up to 95%). Hawkings *et al.* [2] also found elevated DSi concentrations within a sediment-rich plume in the surface waters of a Greenlandic fjord, which directly correlated with salinity. This is unlikely to be explained solely by mixing between fresh and marine end-member waters. A simple leaching experiment carried out by Hawkings *et al.* [2] showed that ASi in SPM of Greenlandic sediments undergoes dissolution in sterile marine waters, releasing bioavailable DSi. Although the DSi profile observed by Hawkings *et al.* [2] appears fairly unusual compared with other fjords sampled, this observation, combined with elevated ASi dissolution rates in marine water, highlights a role of amorphous silica in fjord and near-coastal Si budgets, and could help to explain at least part of the elevated DSi concentrations within the sediment plume. This is also in agreement with studies which suggest that riverine particulate material dissolves upon arrival to the ocean, as explanation of the Si fluxes and oceanic δ^30^Si_DSi_ composition [138].

The fate of glacially exported Si beyond fjords and in the coastal zone, however, is uncertain. Fjords display complex physical, biological and chemical processes on various spatial and temporal scales [130], which could potentially trap glacially supplied nutrients and limit their impact on biogeochemical cycles beyond these fjords [13]. High nutrient consumption by diatoms within fjord systems is one of the most likely mechanisms that could limit the flux of dissolved nutrients into the open ocean [5,131,139]. It is also likely that high nutrient consumption will trap lighter isotopes in the solid phase, as found in brackish/estuarine diatom fractionation [140], meaning DSi would become increasingly isotopically heavy away from the glacial input source. Surface waters influenced by glacial meltwaters off the coastal shelf seas off southwest Greenland in summer 2017 were found to have low DSi concentrations [141]. The low Si may indicate that the dissolution of glacially derived suspended sediments is exceeded by the Si uptake rate of diatoms, or that the ASi fraction has been exhausted in the fjord environments and therefore the kinetics of the remaining Si would be much slower than the dissolution rates of the ASi, so the SPM may not impact the DSi in the ocean. However, these preliminary data also show that these areas of the coastal ocean have elevated turbidity, indicating that glacial suspended sediments could be reaching the coastal ocean, and could therefore still be a potential source of nutrients after further processing of suspended particles. In addition, remote sensing images reveal phytoplankton blooms extending over 300 km into the Labrador Sea, which coincide with addition of glacial meltwaters in south and southwest Greenland [6,142]. However, the mechanism driving these blooms has yet to be fully elucidated. The input of nutrients from glacial meltwaters, most likely Fe, into the ocean may help to stimulate phytoplankton growth, but the production could be linked more to physical processes, such as the increased stratification and mixed layer light levels, rather than nutrient supply *per se* [6,143]. While freshwater entering the open ocean travels hundreds of kilometres from its origin [6,144,145], further work is needed to fully quantify the fluxes of nutrients associated with this glacial meltwater into the open ocean before we can attempt to make firm conclusions around the impact of glacially derived nutrients on wider biogeochemical cycles.

### (b) Impact on the global Si cycle of light Si isotopes from glacial meltwaters?

Despite the uncertainty surrounding nutrient cycling in fjords, it is clear that glacial meltwaters demonstrably reach the open ocean by mixing across continental shelves and are carried significant distances by boundary currents [141]. If glacially derived Si is entering the downstream Si cycle, we can postulate its impact on our current interpretations of oceanic Si cycling and the isotopic composition of the ocean. Glacial rivers export Si with a significantly lighter δ^30^Si_DSi_ composition than non-glacial rivers, yet few Si models currently include glacial fluxes of Si and until recently assumed the δ^30^Si_DSi_ composition of riverine input into the ocean is relatively constant over longer time periods [17]. This could be problematic considering the potentially important glacial Si flux, with the GrIS alone estimated to be 0.20 (0.06–0.79) Tmol yr^−1^ [2], compared with an estimated global riverine flux of Si of 7.3–8.1 Tmol yr^−1^ [17].

The ‘missing’ Si flux from glacial environments could be especially important when considering climatic change over glacial cycles. The isotopic composition of biogenic silica in marine sediment cores can be used as a palaeo-proxy to infer changes in the past Si cycle. For example, biogenic silica isotopic records from the Last Glacial Maximum (LGM) to present day reveal an increase in δ^30^Si of around 0.5–1.0‰, with these findings consistent in the Southern Ocean, North Atlantic and Eastern Equatorial Pacific [17]. These changes have been interpreted as shifts in diatom utilization of silicic acid over glacial--interglacial periods, with lower utilization at the LGM than at present, as increased utilization would enrich surface waters in isotopically heavy Si [146,147]. However, this hypothesis assumes that the riverine input of δ^30^Si into the oceans has been relatively consistent from the LGM to the present day in both flux and δ^30^Si composition. In particular, many previous studies did not consider any impact from glacial meltwaters, which could have a distinct isotopic composition compared with non-glacial rivers [43,44,50].

At the LGM and during deglaciations the oceanic δ^30^Si composition could have been impacted by light glacial δ^30^Si_DSi_ exported from palaeo-ice sheet. These fluxes are likely to have been greatest during Meltwater Pulse 1A and 1B (approx. 14 000–15 000 BP and approx. 11 000 BP, respectively). A simple box model, using the δ^30^Si_DSi_ and δ^30^Si_ASi_ composition of LG in Greenland as an analogue for North American and Eurasian ice sheets, showed that glacial systems may have shifted global oceanic δ^30^Si_DSi_ by 0.06–0.17‰, which accounts for 10–20% of the change in oceanic δ^30^Si_DSi_ since the LGM to present day [50]. In addition, data from a high-frequency sponge spicule core record close to Iceland show variability in δ^30^Si composition of up to −0.6‰ over 300 years, coinciding with rapid Icelandic Ice Sheet collapse [50]. This rapid decrease in δ^30^Si composition, of such a significant magnitude, suggests an influx of glacial meltwater perturbing the system. A rapid flux of isotopically light DSi would probably have been glacially sourced when considering the light δ^30^Si composition measured across the range of glaciers, including those in Iceland (this study, Georg *et al.* [49] and Opfergelt *et al.* [44]). The Si inventory of the ocean is also expected to be larger during the LGM as a result of glacial fluxes, which could have wider implications for primary productivity and CO_2_ drawdown [148]. A larger Si inventory also has the potential to shift the phytoplankton species assemblages over larger spatial scales [50], with a dominance of diatoms over other primary producers expected, as seen in fjord environments with high Si to nitrate ratios [5].

Clearly there are limitations to using such box models and analogue field sites in reconstructing changes in glacial--interglacial Si budgets. The new δ^30^Si_DSi_ data presented in this review highlights the variation in δ^30^Si_DSi_ composition of glacial meltwaters. While all glacial systems export isotopically light Si compared with the global average, the variations in composition do not follow simple trends with parameters such as catchment size. Future modelling efforts should attempt to incorporate the variability in δ^30^Si_DSi_ and δ^30^Si_ASi_ of ice sheet discharge, as well as the fate of ASi in marine waters, which may require further investigation into the temporal changes in Si isotopic composition of glacial meltwaters.

Nevertheless, the modelling study by Hawkings *et al.* [50] highlights the importance of improving our understanding of glacial fluxes and their role in wider biogeochemical cycles. Improving our knowledge of past climatic events will also enable us to model possible future scenarios more robustly, especially considering the increasing role that the glaciers and ice sheets will have upon these biogeochemical cycles under future warming scenarios.

## 5. Summary and suggestions for future progress

Our knowledge of glacial environments, particularly subglacial systems, has increased significantly over the past 20 years, enabling greater insight into the hydrology of glaciers and ice sheets and how they may influence global biogeochemical cycles. At a time when glacial meltwater fluxes are expected to increase as a result of global climatic change [26,149,150], it is extremely important that we understand the chemical and physical processes occurring, so that we can make robust predictions of downstream biogeochemical response, including biological production in the future.

The role of glaciers and ice sheets in nutrient cycling, while much debated for the global scale, is likely to be regionally significant, and fluxes are likely to increase as ice mass loss accelerates. Glacial environments may impact regional Si cycles through fluxes of DSi and dissolvable ASi. However, the subglacial processes that are driving the formation of these silica phases need to be better understood and constrained. There are outstanding uncertainties that require attention in the future to ensure we develop a greater understanding of how glacial environments impact Si cycling, in terms of both Si fluxes and δ^30^Si composition. We therefore propose that, as a community, we should address the following issues.

1. Ensure consistency between sampling methods in glacial environments, especially considering the potential issues from fine-grained glacial flour, colloids and nanoparticulates. A standard protocol for sampling in glacial environments, particularly to measure isotope compositions, would be beneficial to ensure data can be compared between studies and a glacial database be created.
2. Continue to characterize the δ^30^Si composition of glacial meltwaters, with an emphasis on capturing longer term temporal variation, so that the drivers of the isotopically light signals can be identified.
3. Continue to improve understanding of subglacial weathering processes, with particular focus on determining the mechanism of ASi formation in glacial environments. This may involve further field measurements, but also the use of novel analytical techniques and laboratory experiments to further understand the subglacial processes driving Si export.
4. Ensure data collected and used in making wider conclusions are representative of a wider range of glacial environments. For example, we are currently lacking studies of Antarctic glaciers in terms of δ^30^Si composition. Also, Si data are currently much more widely available from west Greenland, compared with east Greenland, where the bedrock lithology is vastly different. More data from smaller alpine glaciers should also be considered important, for example Si fluxes and δ^30^Si composition of rapidly melting Himalayan and Patagonian glaciers are currently lacking but may have an important role in wider biogeochemical cycles, especially considering their increased vulnerability to present-day climate warming.
5. Determine recycling of glacial nutrients in fjord systems and the implications of this for open ocean export. This will require a combination of field measurements (from the surface to the benthic environment) and modelling experiments to understand the complex nature of fjord environments.
6. Ensure glacial Si concentrations and δ^30^Si composition are included in the global Si cycle when considering riverine fluxes and that these are applied to biogeochemical models, once we have constrained fluxes and δ^30^Si composition well enough to be confident of the potential errors of modelling studies.

## Supplementary Material

Supplementary Tables associated with "Silicon Isotopes in Arctic and sub-Arctic Glacial Meltwaters: The Role of Subglacial Weathering in the Silicon Cycle"

## Data accessibility

The data supporting this article are available from: https://doi.pangaea.de/10.1594/PANGAEA.902194.
