# Supplementary material for: Silicon isotopes in Arctic and sub-Arctic glacial meltwaters: the role of subglacial weathering in the silicon cycle
Source: Proc Math Phys Eng Sci. 2019 Aug 14;475(2228):20190098. doi: 10.1098/rspa.2019.0098 (PMC6735475; doi:10.1098/rspa.2019.0098)
Supplement: Supplementary Tables associated with "Silicon Isotopes in Arctic and sub-Arctic Glacial Meltwaters: The Role of Subglacial Weathering in the Silicon Cycle" [file rspa20190098supp1.docx]

Supplementary Information for **“Silicon Isotopes in Arctic and sub-Arctic Glacial Meltwaters: The Role of the Subglacial Weathering in the Silicon Cycle”,** authored by Jade E. Hatton, Katharine R. Hendry, Jonathan R. Hawkings, Jemma, L. Wadham, Sophie Opfergelt, Tyler J. Kohler, Jacob C. Yde, Marek Stibal, and Jakub D. Žárský, published in Proceedings of the Royal Society A.

**Supplementary Table 1. Compilation of silicon concentrations and calculated Si yields from a range of glacierized catchments**

|  | Glacier | Year | Location | Mean DSi Concentration (µmol L^-1^) | DSi yield  (ton km^-2^ a^-1^) | Published data |
| --- | --- | --- | --- | --- | --- | --- |
| Greenland | **Kuannersuit** | 2001 | 69°40’ N 53°17’ W | 146 | 25.99 | Yde *et al.* [1] |
|  | **Kulusuk** | 2013 | 65°42 N 38°27’’ W | 6.8 | - | Aciego *et al.* [2] |
|  | **Leverett Glacier (2015)** | 2015 | 67° 06’ N 50° 17’ W | 20.8 | 1.83 | Hatton *et al.* [3] |
|  | **Kiattuut Sermiat (2013)** | 2013 | 61° 20’N 45° 30’ W | 22.2 | 3.54 | Hatton *et al.* [3] |
|  | **Watson River 2007** | 2007 | 67° N 50° W | 35.6 | 0.40 | Yde *et al.* [4] |
|  | **Watson River 2008** | 2008 | 67° N 50° W | 21.4 | 0.19 | Yde *et al.* [4] |
|  | **Watson River 2009** | 2009 | 67° N 50° W | 42.7 | 0.32 | Yde *et al.* [4] |
| Iceland | **Langjökull** | 1996 | 65° 45’ N 19° 59’ W | Not Reported | 14.3 | Gislason *et al.* [5] |
|  | **Hvita-south, Gullfoss** | 1973 | 64° 19’ N 20° 07’ W | 233 | 13.7 | Gislason *et al.* [5], Hodson *et al.* [6] |
|  | **Hvita-west, Kljafoss** | 1973 | ~64° 19’ N 20° 07’ W | 198 | 10.0 | Gislason *et al.* [5], Hodson *et al.* [6] |
| Svalbard | **Scott Turnerbreen** |  | 78°06’ N 15°57’ E | 3.9 | 0.36 | Hodgkins *et al.* [7] |
|  | **Midre Lovenbreen** | 1998 | 78° 52’ N 11° 57’ E | Not Reported | 0.27 | Hodson *et al.* [8] |
|  | **Midre Lovenbreen** | 1999 | 78° 52’ N 11° 57’ E | Not Reported | 0.22 | Hodson *et al.* [8] |
|  | **Austre Brøggerbreen** | 1999 | ~79°N, 12°E | Not Reported | 0.08 | Hodson *et al.* [8] |
|  | **Bayelva Catchment  (Austre Brøggerbreen and Vestre Brøggerbreen)** | 1991 | ~79°N, 12°E | 4.33 | 0.07 - 0.14 | Hodson *et al.* [9] |
|  | **Bayelva Catchment  (Austre Brøggerbreen and Vestre Brøggerbreen)** | 1992 | ~79°N, 12°E | 3.50 | 0.06 - 0.12 | Hodson *et al.* [9] |
| North America | **Worthington Glacier** | 1995 | 61° 10’ N 145° 45’ W | Not Reported | 4.02 | Anderson *et al.* [10] |
|  | **Kennicott Glacier** | 2000 | 61° 30’ N 143° 00’ W | 33 | 4.84 | Anderson *et al.* [11] |
|  | **Lewis River, Barnes Ice Cap** | 1964 | 70° 0’ N 73° 30’ W | Not Reported | 0.09 | Church [12] |
|  | **Berendon Glacier** | 1975 | 56° 14’ N 130° 10’ W | Not Reported | 0.51 - 1.87 | Eyles *et al.* [13] |
|  | **South Cascade Glacier** | 1992 | 48° 21’ N 121° 3’ W | Not Reported | 1.87 | Reynolds and Johnson [14] |
| Himalayas | **Batura Glacier** | 1999 | 35° 44’ N 76° 22’E | 24.9 | 1.12 | Hodson *et al.* [15] |
|  | **Gangotri Glacier** | 2007 | 30°43’ N 78°59’ E | 64 | 8.63 | Srivastava [16], Singh *et al.* [17] |
| Swiss Alps | **Rhone Glacier** | 2000 | 46°35’ N 8°23’ E | Not Reported | 3.96 - 4.83 | Hosein *et al.* [18] |
|  | **Oberaar Glacier** | 2000 | 46°32’ N 8°14’ E | Not Reported | 4.80 | Hosein *et al.* [18] |
|  | **Haut Glacier d’Arolla** | 1990 | 45° 59’N 7° 29’ E | Not Reported | 1.96 - 2.15 | Sharp *et al.* [19] |

**Supplementary Table 2: Sample information for the new data presented in this study.**

| Glacier | Location | Latitude | Longitude | Date | Time | | Temp (°C) | Sample ID |
| --- | --- | --- | --- | --- | --- | --- | --- | --- |
| Disko 6 | Qeqertarsuaq | 69.715833° | -53.441617° | 4/8/2015 | 1600 | 0.1 | | D6 |
| Disko 10 | Qeqertarsuaq | 69.766717° | -53.413400° | 6/8/2015 | 1520 | 0.1 | | D10 |
| Disko 11 | Qeqertarsuaq | 69.784050° | -53.427200° | 6/8/2015 | 1800 | 2.2 | | D11 |
| Disko 13 | Qeqertarsuaq | 69.801817° | -53.375900° | 9/8/2015 | 1700 | 0.1 | | D13 |
| Kuannersuit | Qeqertarsuaq | 69.687500° | -53.291450° | 3/8/2015 | 2030 | 0.8 | | KG |
| Nansenbreen | Svalbard | 78.353145° | 14.075243° | 2/8/2016 | 1345 | 0.9 | | Nan |
| Sefströmbreen | Svalbard | 78.719740° | 14.374894° | 4/8/2016 | 2030 | 0.8 | | Sef |
| Ebbabreen | Svalbard | 78.726805° | 16.794599° | 6/8/2016 | 1630 | 0.6 | | Ebba |
| Langjökull | Iceland | 64.496778° | -20.227667° | 15/8/2016 | 1400 | - | | Lang |
| Sólheimajökull | Iceland | 63.534833° | -19.352194° | 16/8/2016 | 1500 | - | | Sol |
| Skaftafellsjökull | Iceland | 64.028667° | -16.932667° | 17/8/2016 | 1100 | - | | Skaf |
| Eyjabakkajökull | Iceland | 64.666250° | -15.723694° | 18/8/2016 | 1500 | - | | Eyja |
| Drangajökull | Iceland | 66.117611° | -22.287750° | 20/8/2016 | 1400 | - | | Drang |
| Styggedalsbreen | Norway | 61.488306° | 7.880444° | 22/9/2016 | 1400 | 0.7 | | Sty |
| Austerdalsbreen | Norway | 61.588500° | 6.995333° | 24/9/2016 | 1400 | 0.3 | | Aus |
| Bøverbreen | Norway | 61.556694° | 8.049500° | 25/9/2016 | 1400 | 0.7 | | Bov |
| Herbert | Alaska | 58.539120° | -134.684540° | 27/6/2017 | 1230 | 0.3 | | HE |
| Mendenhall | Alaska | 58.403990° | -134.581670° | 30/6/2017 | 1030 | 3.6 | | ME |
| Lemon | Alaska | 58.364320° | -134.478740° | 29/6/2017 | 1115 | 5.3 | | LE |
| Eagle | Alaska | 58.528640° | -134.805680° | 29/6/2017 | 1400 | 3.7 | | EA |
| Watson River | Greenland | 67.007460° | -50.680240° | 12/9/2017 | 1000 | 0.3 | | Wat |

**Supplementary Table 3: Summary of hydrochemical and isotopic results from the range of glaciers presented in this study.** Area refers to the glacierised part of the catchment, rather than the total catchment area. EC = Electrical Conductivity, D:M = Divalent ion (Ca^2+^ +Mg^2+^) : Monovalent ion (K^+^:Na^+^) ratio, ASi = Amorphous silica concentration, DSi = Dissolved silicon concentration.

| Sample | pH | EC  (µS cm^-1^) | D:M  (µeq) | DSi  (µmol l^-1^) | δ^30^Si_DSi_  (‰) | ASi  (%) | δ^30^Si_Asi_  (‰) | Area (km^2^) |
| --- | --- | --- | --- | --- | --- | --- | --- | --- |
| D6 | 7.2 | 7.9 | 1.66 | 15.8 | 0.22 | 0.49 | -0.55 | 1.50 |
| D10 | 8.7 | 7.7 | 4.65 | 23.3 | -0.15 | 0.49 | -0.48 | 7.00 |
| D11 | 6.9 | 9.9 | 1.38 | 7.92 | -0.36 | 0.43 | -0.61 | 9.70 |
| D13 | 7.5 | 9.0 | 0.54 | 34.8 | 0.24 | 0.50 | ­­­­-0.67 | 18.0 |
| KG | 8.7 | 13.8 | 0.95 | 33.8 | -0.13 | - | - | 103 |
| Nan | 7.2 | 70 | 32.16 | 4.56 | -0.07 | 0.12 | -0.66 | 38.1 |
| Sef | 8.3 | 108 | 143.7 | 3.31 | 0.18 | 0.09 | -0.27 | 133 |
| Ebba | 7.6 | 112 | 18.1 | 3.03 | 0.16 | 0.10 | -0.71 | 1.68 |
| Lang | 8.3 | 28.3 | 1.45 | 49.4 | -0.58 | 0.23 | -0.10 | 131 |
| Sol | 8.8 | 48.0 | 0.88 | 94.8 | 0.78 | 2.10 | -0.06 | 55.1 |
| Skaf | 9.4 | 30.5 | 0.85 | 39.3 | -0.09 | 0.55 | -0.18 | 90.5 |
| Eyja | 8.2 | 5.6 | 1.91 | 16.5 | -0.51 | 1.72 | -0.05 | 130 |
| Drang | 8.5 | 6.5 | 0.83 | 13.6 | -0.14 | 0.59 | -0.36 | 41.9 |
| Sty | 7.5 | 3.0 | 3.75 | 10.7 | -0.09 | 0.66 | -0.31 | 2.06 |
| Aus | 6.7 | 25 | 5.28 | 26.6 | 0.63 | 0.28 | -0.34 | 20.9 |
| Bov | 6.1 | 2.0 | 1.11 | 4.95 | 0.53 | 0.13 | -0.54 | 9.58 |
| HE | 7.7 | 21 | 4.22 | 13.5 | 0.49 | 0.34 | -0.62 | 61.2 |
| ME | 8.0 | 26 | 3.98 | 17.4 | 0.59 | 0.44 | -0.54 | 109 |
| LE | 7.8 | 38 | 7.56 | 24.6 | 0.46 | 0.14 | -0.86 | 9.53 |
| EA | 8.1 | 18 | 3.33 | 18.4 | 0.33 | 0.64 | - | 40.5 |
| Wat | 8.5 | 36 | 0.47 | 33.2 | 0.31 | 1.47 | -0.30 | 6100 |

References

[1] Yde, J.C., Tvis K. N. & Nielsen, O.B. 2005 Glacier hydrochemistry, solute provenance, and chemical denudation at a surge-type glacier in Kuannersuit Kuussuat, Disko Island, West Greenland. *Journal of Hydrology* **300**, 172-187. (doi:<https://doi.org/10.1016/j.jhydrol.2004.06.008>).

[2] Aciego, S.M., Stevenson, E.I. & Arendt, C.A. 2015 Climate versus geological controls on glacial meltwater micronutrient production in southern Greenland. *Earth and Planetary Science Letters* **424**, 51-58. (doi:<https://doi.org/10.1016/j.epsl.2015.05.017>).

[3] Hatton, J.E., Hendry, K.R., Hawkings, J.R., Wadham, J.L., Kohler, T.J., Stibal, M., Beaton, A.D., Bagshaw, E.A. & Telling, J. 2019 Investigation of subglacial weathering under the Greenland Ice Sheet using silicon isotopes. *Geochim. Cosmochim. Acta*. (doi:<https://doi.org/10.1016/j.gca.2018.12.033>).

[4] Yde, J.C., Knudsen, N.T., Hasholt, B. & Mikkelsen, A.B. 2014 Meltwater chemistry and solute export from a Greenland Ice Sheet catchment, Watson River, West Greenland. *Journal of Hydrology* **519**, 2165-2179. (doi:10.1016/j.jhydrol.2014.10.018).

[5] Gislason, S.R., Arnorsson, S. & Armannsson, H. 1996 Chemical weathering of basalt in Southwest Iceland; effects of runoff, age of rocks and vegetative/glacial cover. *American Journal of Science* **296**, 837-907. (doi:10.2475/ajs.296.8.837).

[6] Hodson, A., Tranter, M. & Vatne, G. 2000 Contemporary rates of chemical denudation and atmospheric CO2 sequestration in glacier basins: an Arctic perspective. *Earth Surface Processes and Landforms* **25**, 1447-1471. (doi:doi:10.1002/1096-9837(200012)25:13<1447::AID-ESP156>3.0.CO;2-9).

[7] Hodgkins, R., Tranter, M. & Dowdeswell, J.A. 1997 Solute provenance, transport and denudation in a high arctic glacierized catchment. *Hydrological Processes* **11**, 1813-1832. (doi:doi:10.1002/(SICI)1099-1085(199711)11:14<1813::AID-HYP498>3.0.CO;2-C).

[8] Hodson, A.J., Mumford, P.N., Kohler, J. & Wynn, P.M. 2005 The High Arctic glacial ecosystem: new insights from nutrient budgets. *Biogeochemistry* **72**, 233-256. (doi:10.1007/s10533-004-0362-0).

[9] Hodson, A., Tranter, M., Gurnell, A., Clark, M. & Hagen, J.O. 2002 The hydrochemistry of Bayelva, a high Arctic proglacial stream in Svalbard. *Journal of Hydrology* **257**, 91-114. (doi:<https://doi.org/10.1016/S0022-1694(01)00543-1>).

[10] Anderson, S.P., Drever, J.I. & Humphrey, N.F. 1997 Chemical weathering in glacial environments. *Geology* **25**, 399-402. (doi:10.1130/0091-7613(1997)025<0399:cwige>2.3.co;2).

[11] Anderson, S.P., Longacre, S.A. & Kraal, E.R. 2003 Patterns of water chemistry and discharge in the glacier-fed Kennicott River, Alaska: evidence for subglacial water storage cycles. *Chemical Geology* **202**, 297-312. (doi:<https://doi.org/10.1016/j.chemgeo.2003.01.001>).

[12] Church, M. 1974 On the Quality of Some Waters on Baffin Island, Northwest Territories. *Canadian Journal of Earth Science* **11**, 1676-1688.

[13] Eyles, N., Sasseville, D.R., Slatt, R.M. & Rogerson, R.J. 1982 Geochemical denudation rates and solute transport mechanisms in a maritime temperate glacier basin *Canadian Journal of Earth Science* **19**, 1570-1581.

[14] Reynolds, R.C. & Johnson, N.M. 1972 Chemical weathering in the temperate glacial environment of the Northern Cascade Mountains. *Geochim. Cosmochim. Acta* **36**, 537-554. (doi:<https://doi.org/10.1016/0016-7037(72)90074-9>).

[15] Hodson, A., Porter, P., Lowe, A. & Mumford, P. 2002 Chemical denudation and silicate weathering in Himalayan glacier basins: Batura Glacier, Pakistan. *Journal of Hydrology* **262**, 193-208. (doi:<https://doi.org/10.1016/S0022-1694(02)00036-7>).

[16] Srivastava, D. 2012 Status Report on Gangotri Glacier In *Himalayan Glaciology*

*Technical Report* (New Delhi, Science and Engineering Research Board, Department of Science and Technology.

[17] Singh, V.B., Ramanathan, A., Pottakkal, J.G., Sharma, P., Linda, A., Azam, M.F. & Chatterjee, C. 2012 Chemical characterisation of meltwater draining from Gangotri Glacier, Garhwal Himalaya, India. *Journal of Earth System Science* **121**, 625-636. (doi:10.1007/s12040-012-0177-7).

[18] Hosein, R., Arn, K., Steinmann, P., Adatte, T. & F⊙llmi, K.B. 2004 Carbonate and silicate weathering in two presently glaciated, crystalline catchments in the Swiss Alps. *Geochim. Cosmochim. Acta* **68**, 1021-1033. (doi:<https://doi.org/10.1016/S0016-7037(03)00445-9>).

[19] Sharp, M., Tranter, M., Brown, G.H. & Skidmore, M. 1995 Rates of chemical denudation and CO2 drawdown in a glacier-covered alpine catchment. *Geology* **23**, 61-64.
